# A Combined Workplace Intervention Including Exercise and Nutrition for Health Promotion in Obese Office Workers: A Randomized Controlled Study

**DOI:** 10.3390/jfmk11040418

**Published:** 2026-10-13

**Authors:** Konstantina Karatrantou, Elisavet Efthymiou, Christos Batatolis, Konstantinos Pappas, Theodora Vasilopoulou, Anastasia Melissopoulou, Vassilis Gerodimos

**Affiliations:** Department of Physical Education and Sports Science, University of Thessaly, 42100 Trikala, Greece; elisefth@gmail.com (E.E.); batatoli@uth.gr (C.B.); ntinospappas7@gmail.com (K.P.); vasilopoulouphys@gmail.com (T.V.); nutrition4health2021@gmail.com (A.M.)

**Keywords:** occupational setting, wellness, physical activity, functional capacity, dietary habits, Mediterranean diet, hydration

## Abstract

**Background:** During the last decades, the office workplace setting has increasingly become an inherently “obesogenic” environment, promoting unhealthy behaviors. The main objective of this study was to investigate the efficacy of a 6-month workplace combined (exercise and nutrition) intervention in obese office workers, using a randomized controlled study. An additional objective of this study was to evaluate the individual responses per participant following the program. **Methods:** In total, 36 obese office workers (BMI: ≥30) were randomly assigned to two equal groups: intervention (IG; age: 48.06 ± 6.60 yrs; BMI: 33.57 ± 1.58 kg/m^2^) and control (CG; age: 47.95 ± 5.95 yrs; BMI: 33.54 ± 1.74 kg/m^2^). The IG participated in a daily supervised 6-month workplace program (during working hours), including (a) exercise (flexibility, balance, strength, and aerobic) and (b) counseling and educational activities for healthy diet and hydration. The CG did not participate in any intervention. Before and after the 6-month period, the following were measured: (a) health indices (body fat, body circumferences, blood pressure, respiratory function, and lipidemic profile), (b) functional capacity indices (flexibility, balance, handgrip strength, and cardiovascular fitness), and (c) adherence to the Mediterranean diet (MedDietScore) and daily water consumption. **Results:** All the participants of the IG reduced body mass, body fat, and circumferences (−4.47 to −18.95%), blood pressure (−4.08 to −46.15%), lipidemic profile indicators (−2.50 to −212.67%), and heart rate recovery (−9.62 to −57.33%), while increased respiratory function (1.82–20.26%), flexibility (5.13–50%), balance (15.38–88.83%), handgrip strength (5–25%), MedDietScore (12.5–56%), and water consumption (23.08–50%). In CG, all aforementioned indices remained unchanged (*p* = 0.16–0.99). **Conclusions:** A combined workplace intervention (exercise and nutrition) constitutes an effective approach for health promotion in obese office workers.

## 1. Introduction

The growing prevalence of obesity among workers constitutes a serious public health problem, driven by the environmental and behavioral constraints of the modern workplace setting (progressive transition from manual labor to desk-based roles) [1,2]. Recent data report that the workplace setting may act as an “obesogenic” environment that significantly increases the risk of weight gain, promotes unhealthy behaviors (prolonged sedentary time, bad eating habits, smoking, alcohol consumption, etc.), and causes high psychosocial stress levels [3,4]. The term “obesogenic” environment, which first appeared in the 1990s [5], has piqued the interest of the global scientific literature in searching for effective strategies and interventions to promote a healthy lifestyle [6] in different populations. This is of crucial importance, especially in the office work population, where several studies all over the world showed high combined prevalence of overweight and obesity (from 60% to 77.7%) [7,8] and prolonged exposure in sedentary positions during the workday (>78% of their time) [9].

The high prevalence of obesity in conjunction with the adoption of an unhealthy lifestyle may negatively affect not only workers’ health and quality of life, but also their work productivity and the economy in general [10,11]. Indeed, a previous study from Dall et al. [11] estimated that the combined economic burden of overweight and obesity among workers is $425.5 billion, including medical expenses, absenteeism, disability payments, compensation payments, and reduced productivity. Taking all the above into consideration, the World Health Organization (WHO), using the Global Action Plan (2018–2030), recognizes the workplace as a critical setting for increasing physical activity and promoting healthy lifestyle interventions, directly targeting to reduce obesity, non-cumulative diseases, and sedentary behavior.

Several studies have examined the efficiency of various interventions (i.e., exercise, nutrition, or combined exercise and nutrition) for health promotion in obese individuals [12,13,14,15,16,17,18,19]; however, the information regarding interventions in the workplace setting in obese office workers is limited. More specifically, it should be mentioned that most of previous studies that implemented intervention for obese office workers (a) focused mainly on intervention that included sit to stand, treadmill, and/or pedal workstations to increase physical activity and decrease sedentary time [20,21,22,23,24,25,26,27]; (b) assessed limited indicators of health and/or physical fitness; (c) used a mixed sample of overweight-obese individuals [20,21,22,23,24,25,27,28,29,30]; and/or (d) reported limited or conflicting results (either improved or no change on the evaluated parameters) [21,26,27,30]. For example, Phaswana et al. [26] examined the efficacy of a 12-week height-adjustable sit-to-stand intervention on sedentary behavior and cardiometabolic health outcomes among overweight-obese office-based workers in South Africa, reporting encouraging results (with however small—trivial effect sizes). Another study of Ramli et al. [30] investigated the efficiency of a 6-month health program (including weekly unsupervised exercise sessions and monthly dietary/health education sessions) among overweight and obese office workers, demonstrating significant effects on body fat, maximum oxygen uptake, curl-up, and sit-and-reach test. In the above study of Ramli et al. [30], no effects were observed on body mass, push-up test, self-perceived level of physical activity, and behavior toward exercise. To the best of our knowledge, no previous study has examined a supervised comprehensive workplace intervention, including exercise (flexibility, balance, strength, and aerobic capacity) and nutrition for health promotion in a clearly obese sample of office workers, evaluating a wide range of indicators.

Therefore, the main objective of this study was to design, implement, guide, and evaluate the efficacy of a 6-month daily supervised workplace (during the working hours) combined intervention program, including exercise (flexibility, balance, strength, and aerobic capacity) and nutrition on selected health (body mass, body fat and circumferences, blood pressure, respiratory function, and lipidemic profile); (b) functional capacity indices (flexibility, static and dynamic balance, handgrip strength, and cardiovascular fitness); and (c) dietary habits (adherence to the Mediterranean diet and daily water consumption amount) of obese office workers. Another important objective of this study was to investigate the individualized responses per participant following the 6-month workplace intervention program. This is of fundamental importance since the individual characteristics of the participants may affect the efficiency of the intervention program and the generalization of study results in the whole population. This notion has been strengthened by the results of a previous study [31] that was implemented in health professionals, and the analyses for the average of the sample showed limited or non-homogeneous changes following an educational program, while supplementary individual analyses highlighted substantial changes in perceptions, attitudes, and self-efficacy among some of the participants. The authors of the above study [31] mentioned that the effect of the intervention varied significantly depending on individual factors, such as initial skill level, professional experience, and degree of active participation, demonstrating the importance of complementary interpretation through individual data where the scientific literature is limited.

It was hypothesized that the 6-month combined workplace intervention, including exercise and nutrition, would reduce body mass, body fat, body circumferences, blood pressure, and lipidemic profile indices (total cholesterol, low-density lipoprotein, and triglycerides) and improve functional capacity indices as well as adherence to the Mediterranean diet in all the participants of the intervention group. It was also hypothesized that the above parameters would remain unchanged or worsen in all the participants of the control group. It is expected that the combined workplace intervention, including exercise and nutrition, will be used as a “best practice” in the specific office environments to improve health-related quality of life in workers. In the present study, we decided to use a simple exercise program with low-cost equipment that may be implemented after specific education (using the appropriate exercise material, such as photos and videos) by the workers themselves, with sporadic supervision. Finally, the appropriate educational material regarding proper nutrition and hydration that will be designed during this study could be used by the workers for further health promotion after the termination of the intervention program.

## 2. Materials and Methods

### 2.1. Participants

**Figure 1:**
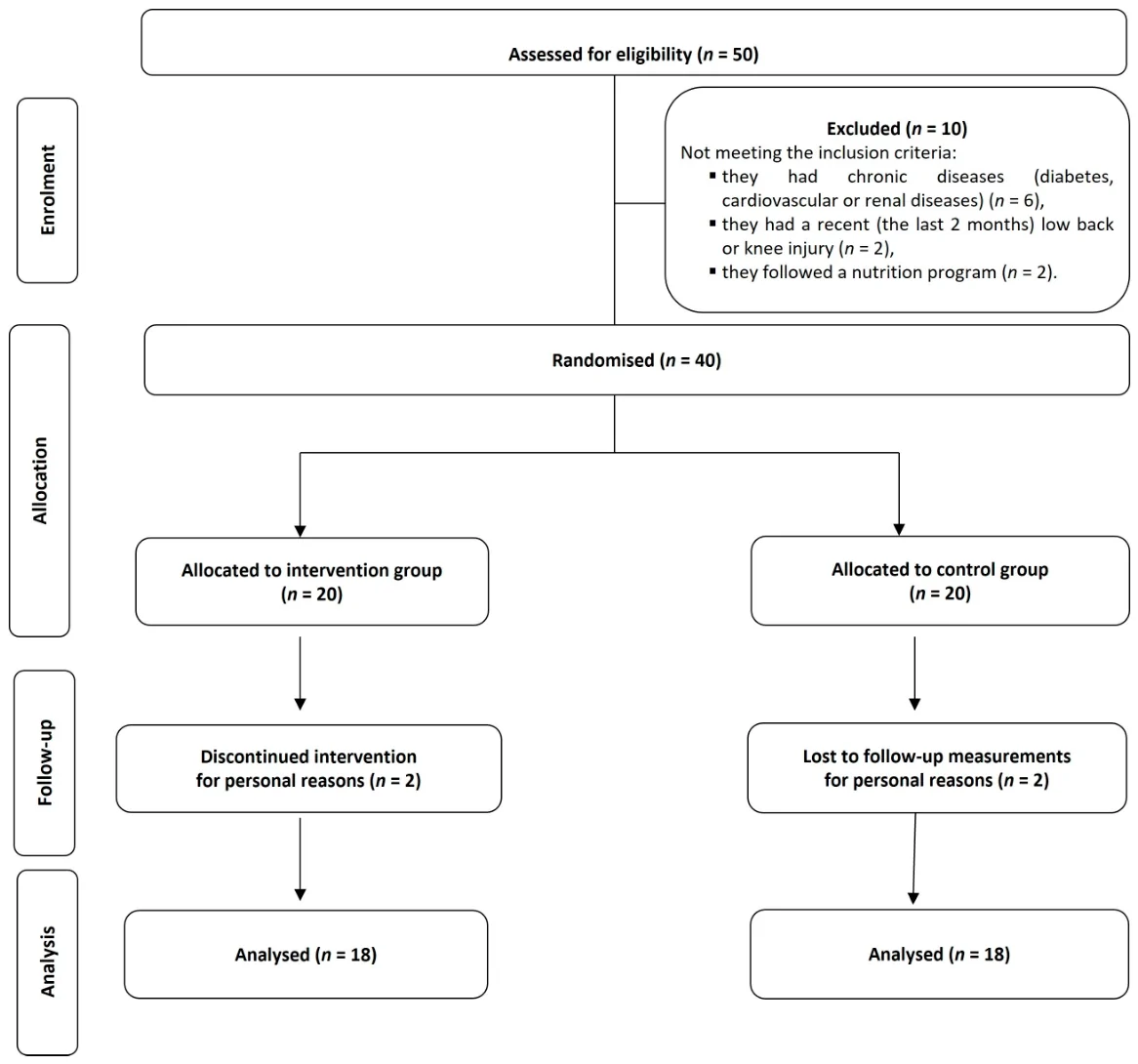
Flow chart of the progress through the four phases of a randomized study: (1) enrollment, (2) allocation, (3) follow-up, and (4) analysis.

In total, 50 male and female obese office workers, ages 34–55 years old, expressed interest in participating after receiving announcements from the research team. Announcements were made in four workplaces in Western Thessaly. The participants were assessed for eligibility according to six inclusion criteria. In more detail, the participants (1) should have at least 8 h per day in the office, (2) should have BMI value ≥ 30 kg/m^2^, (3) should not have chronic diseases (physical or mental), (4) should not use any medication, (5) should not have injuries (in lower or upper body) the last 6 months, and (6) should not participate in organized exercise programs or nutrition intervention during the last 6 months. Following the screening of eligibility, 10 of the 50 office workers who were assessed were excluded from the study because they did not meet some of the inclusion criteria (Figure 1).

Thus, 40 office workers volunteered to participate in the present study, and following baseline measurements, they were randomly assigned to either an intervention group (IG) or a control group (CG). It should be mentioned that two participants from each group dropped out of the study, and consequently, the final number of participants was 36 (Figure 1). The participants who did not complete the intervention or did not participate in the second measurements were not included in the statistical analysis.

Prior to the start of the study, the demographic, anthropometric, and working characteristics of the participants (Table 1) were assessed using the ACSM health history questionnaire [32].

### 2.2. Intervention

The IG participated in a supervised 6-month combined intervention program (exercise and nutrition), which took place inside the workplace settings during working hours.

#### 2.2.1. Exercise Intervention

The IG participated every working day (5 days/week) in a 6-month supervised combined chair-based exercise program (120 training sessions; 25–40 min/day), including chair-based seated and chair-assisted standing exercises to improve flexibility, balance, strength, and aerobic capacity. The frequency of weekly exercise goals was altered throughout the 6-month exercise intervention, according to the recommendations of the ACSM [32,33]. Two indicative weekly tables with the exercise goals of the intervention program are analytically presented in Table 2.

The exercise program was implemented in small groups (4–5 participants per group), using a stable chair as the basic exercise equipment as well as portable auxiliary exercise equipment such as a Pilates mini ball, a pair of silicone hand therapy balls, a pair of hand grippers, and an exercise band for each participant. Each training session consisted of a 3–5 min warm-up, a 19–34 min main part of the program, and a 3–5 min cooldown. The training load gradually increased during the 6-month intervention program, according to the recommendation of the ACSM [32,33]. The training contents and variables of the exercise program are analytically presented in Table 3.

Two indicative training units of the 6-month workplace exercise program are analytically presented in Supplementary Tables S1 and S2.

#### 2.2.2. Nutrition and Hydration Intervention

**Figure 2:**
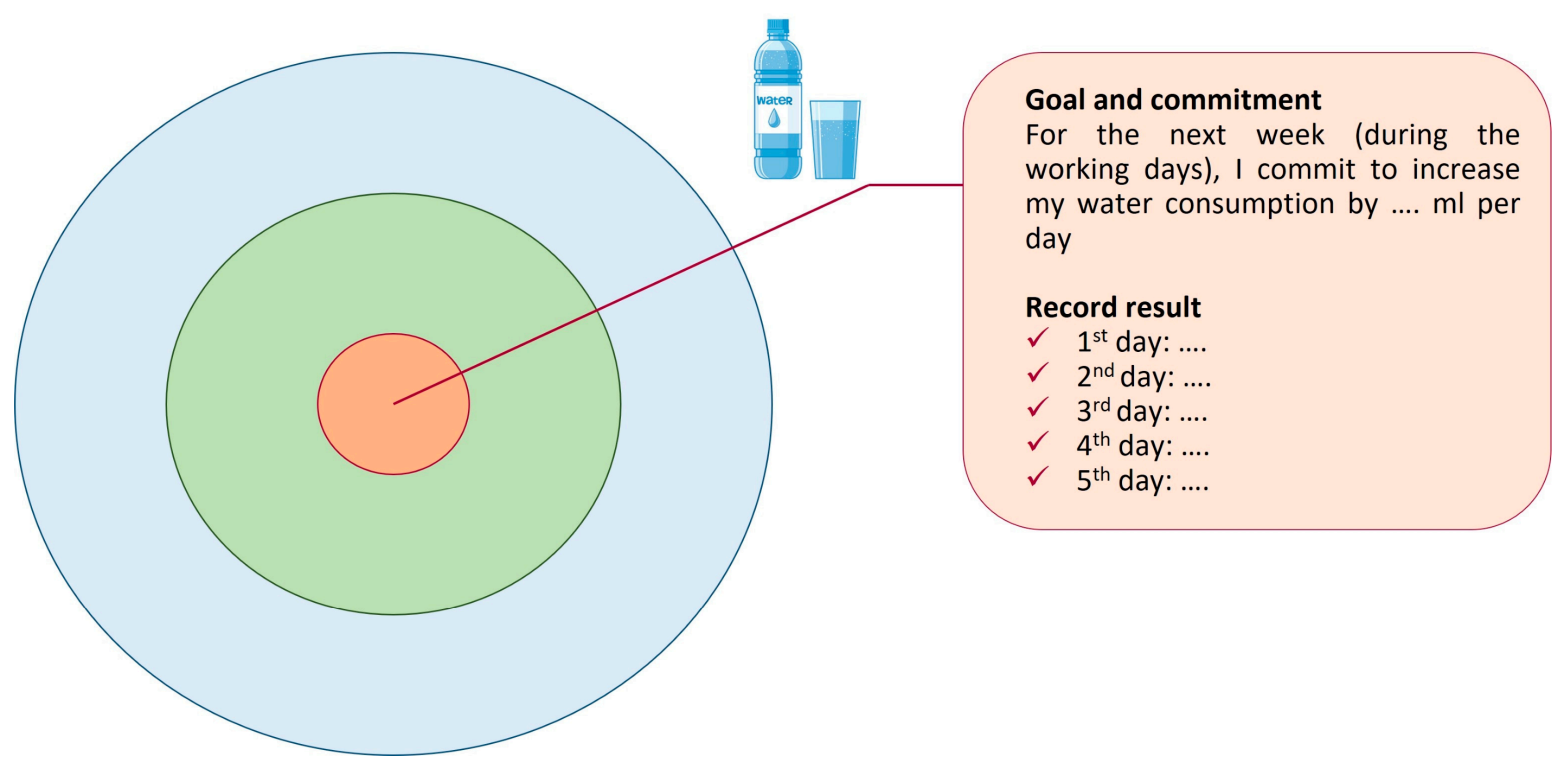
Weekly indicative goal-setting card for water consumption. The three circles in Figure 2 represent the target levels (from long-term to short-term): the large blue circle represents the 2-week target, the green circle represents the weekly target, and the orange circle represents the target for each working day of the week.

The intervention regarding healthy diet and proper hydration included 12 sessions (one session every 2 weeks) with the two nutritionists of the research team (Table 4). It should be mentioned that for the implementation of the intervention were used specifically designed educational material such as (a) presentations regarding the importance of healthy diet and proper hydration; (b) educational leaflets with tips for healthy diet based on the principles of the Mediterranean diet (appropriate frequency consumption of basic foods and beverages) as previously described by previous investigators [34,35] as well as indicative recipes for healthy snacks at the workplace; (c) goal-setting cards for healthy options regarding nutrition and water consumption, which were adjusted periodically every two weeks during the sessions of nutrition and hydration intervention (Figure 2); and (d) free application for proper hydration Waterful https://waterfulapp.com/?from=AppAgg.com (accessed on 10 July 2026), which all the participants of the IG downloaded to their mobile phone during the study.

### 2.3. Measurements

**Figure 3:**
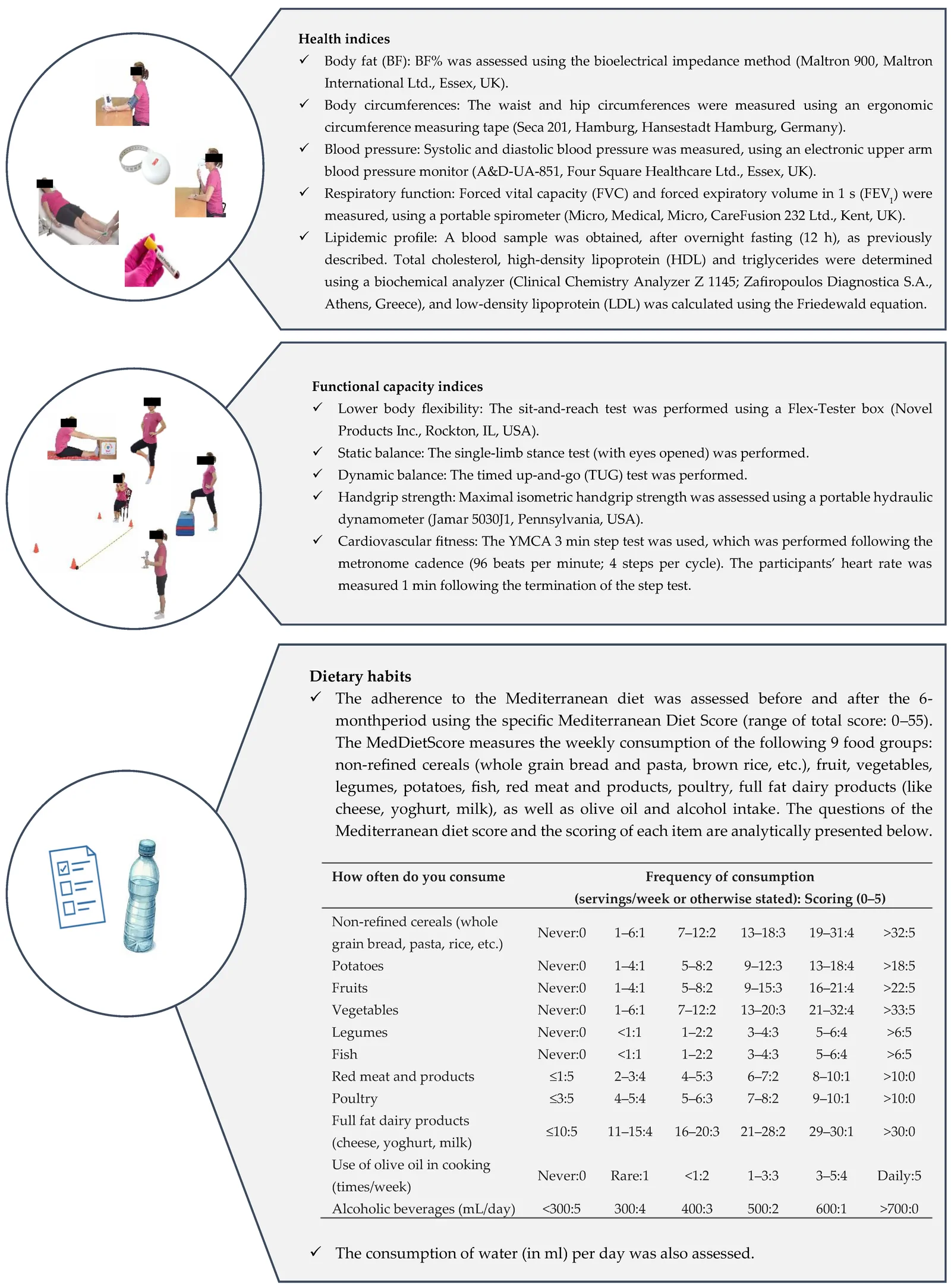
Health, functional capacity, and dietary habits measurements before and after the 6-month time period.

Before and after the completion of the 6-month workplace intervention program, selected health and functional capacity indices were assessed (Figure 3) [32,36,37,38,39,40,41], using widely used, and reliable, tests that have shown high test-retest reliability (ICC = 0.95–0.98; SEM = 0.76–1.22; SEM% = 2.6–4) in office workers [7]. Furthermore, the adherence to the Mediterranean diet was assessed before and after the 6-month time period using a specific questionnaire [34,35].

### 2.4. Procedure

The present study was conducted according to the Declaration of Helsinki and approved by the Ethics Committee of the University of Thessaly. This study was registered at ClinicalTrials.gov (Identifier: NCT07603141).

Prior to the start of the study, the participants (a) were informed about the experimental procedures and possible risks, (b) were familiarized with the testing and exercise procedures, and (c) signed an informed consent form. Following the initial information and familiarization procedure, the baseline measurements were performed inside the workplace setting (during working hours) by the same investigators and under the same conditions. The sequence of measurements was (1) health indices, (2) functional capacity indices (flexibility, balance, strength, and cardiovascular fitness), and (3) dietary habits questionnaire. Before the commencement of the functional capacity tests, the participants performed a standardized 8-minute warm-up protocol (3-minute chair-based aerobic dance and 5 minutes of static and dynamic stretching for lower and upper body). Following the baseline measurements, the participants were randomly assigned to two equal groups: the IG and the CG. In the present study, a parallel randomized controlled design was used to investigate the efficiency of the 6-month workplace combined intervention program (exercise and nutrition) on health, functional capacity, and dietary habits indices in obese office workers (Figure 1; Supplementary Table S3). Specifically, a computer-generated list of random numbers was used for the allocation of the participants in one of the two groups (IG or CG). The main investigator and the outcome assessors were blinded to the group allocation during the study, and the participants were requested not to discuss their intervention with either the main investigator or the assessors.

During the study, the IG participated in a daily 6-month (5 times per week) combined intervention program consisting of exercise and nutrition, while the CG did not perform any intervention during the 6 months. It would be important to mention that no adverse effects or injuries were reported during the 6-month exercise program. The exercise program of the intervention was supervised by three exercise instructors of the research team, and special exercise attendance books were filled in to confirm the participants’ exercise adherence (all the participants of the study completed 120 exercise sessions in total), while the nutrition part of the intervention was supervised by the two nutritionists of the research team. After the end of the 6-month period, the baseline measurements were repeated in the same order and at the same time of day. It should also be mentioned that throughout the 6 months, the participants of the IG did not alter their daily habits outside the workplace and did not undergo any intervention other than the specific study intervention implemented at the workplace.

### 2.5. Statistical Analysis

All statistical analyses were performed using the IBM SPSS Statistics v.31 software (IBM Corporation, Armonk, New York, NY, USA), and the results are presented as means ± standard deviations. Before the commencement of the study, a power analysis, using software package GPower v. 3.1 for statistical test “ANOVA: Repeated measures, within-between interaction (2 groups and 2 measures)” and effect size 0.30 (a err prob = 0.05) was performed. The statistical power analysis indicated that a total sample of 32 participants (16 participants in each group) would yield adequate power (0.90). Therefore, the final sample of the present study was 36 participants (18 participants per group). The normality of the data was examined using the Shapiro–Wilk test, where all variables followed the normal distribution. Two-way analyses of variances (two-way ANOVAs) [2 groups (IG and CG) × 2 time points (pre- and post-intervention)] with repeated measures on the time point factor and Sidak pairwise comparisons were applied to locate the significantly different means within and between groups. Independent *t*-tests were also used to compare the percentage change (from pre- to post-measurement) between the two groups (IG and CG). The magnitude of the difference between measurements and groups was examined using Cohen’s *d* effect sizes (ES). The level of significance for all statistical analyses was set at *p* < 0.05.

## 3. Results

### 3.1. Health Indices

#### 3.1.1. Body Fat and Body Circumferences (Waist and Hip Circumferences)

Repeated measures analyses of variances (ANOVAs) showed significant “group × time” interaction effects on the body mass (*F*_1,34_ = 269.32; *p* < 0.001), percentage of body fat (*F*_1,34_ = 378.59; *p* < 0.001), waist (*F*_1,34_ = 180.17; *p* < 0.001), and hip (*F*_1,34_ = 167.54; *p* < 0.001) circumferences. Specifically, in the IG, post-training values on the body mass (*p* < 0.001; Cohen *d* = 0.46, moderate effect size), percentage of body fat (*p* < 0.001; Cohen *d* = 2.97 large effect size), waist circumference (*p* < 0.001; Cohen *d* = 1.17 large effect size), and hip circumference (*p* < 0.001; Cohen *d* = 1.87 large effect size) were significantly lower compared to the pre-training measurements (Table 5). In CG, the aforementioned variables remained unaffected after 6 months (*p* = 0.11–0.41). Comparisons between groups revealed that pre-training body mass, body fat, and body circumferences values were not different between groups (*p* = 0.67–0.99), but all post-training values were significantly lower in IG vs. CG (*p* < 0.05–0.001; Cohen *d* = 1.27–3.25 large effect size). Independent *t*-tests showed that the percentage changes (from baseline to final measurements) in body mass, body fat, and body circumferences were significantly greater in IG than in CG (*p* < 0.001).

**Figure 4:**
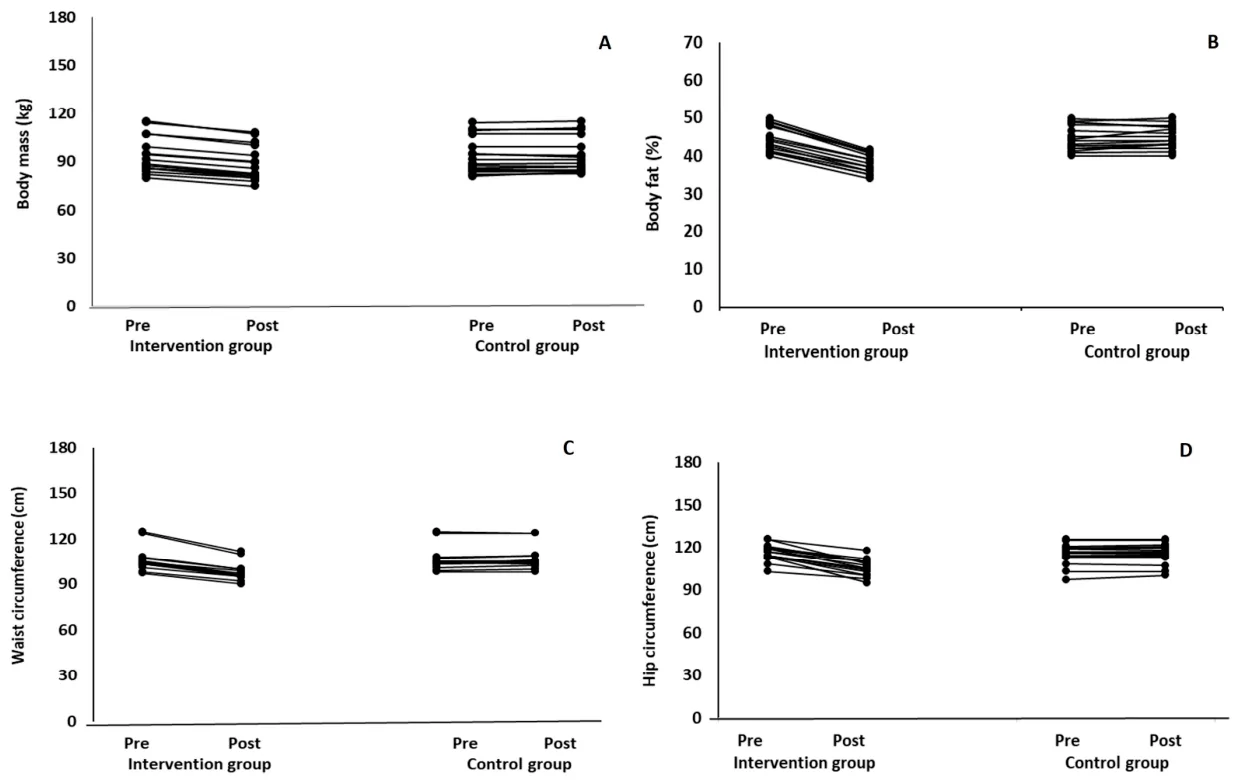
Individualized changes pre- and post-training in body mass (**A**), body fat (**B**), waist circumference (**C**), and hip circumference (**D**) in the intervention and control groups.

When the results were analyzed and presented individually (Figure 4), we observed that all the participants in the IG, following the intervention program, reduced body mass, body fat, and body circumference values. The range of reduction was from −4.47 to −6.98% for the body mass, from −5 to −13% for the percentage of body fat, from −6.19 to −12.84% for the waist circumference, and from −5.10 to −18.95% for the hip circumference, depending on the participant. In the CG, the individualized analyses indicated conflicting results (some participants reported no change, while others showed either a slight increase or decrease) following the 6 months.

#### 3.1.2. Blood Pressure (Systolic and Diastolic)

Repeated measures analyses of variances indicated significant “group × time” interaction effects on the systolic blood pressure (*F*_1,34_ = 21.53; *p* < 0.001) and on the diastolic blood pressure (*F*_1,34_ = 33.84; *p* < 0.001). Specifically, in the IG, post-training values on the systolic blood pressure (*p* < 0.001; Cohen *d* = 1.05 large effect size) and on the diastolic blood pressure (*p* < 0.001; Cohen *d* = 0.91 large effect size) were significantly lower compared to the pre-training measurements (Table 6). In CG, the above variables remained stable after 6 months (*p* = 0.76–0.85). Comparisons between groups revealed that pre-training blood pressure values (systolic and diastolic) were not different between groups (*p* = 0.71–0.94), but all post-training values were significantly lower in IG vs. CG (*p* < 0.001; Cohen *d* = 0.97–0.99 large effect size). Independent *t*-tests showed that the percentage changes (from baseline to final measurements) in blood pressure were significantly greater in IG than in CG (*p* < 0.001).

**Figure 5:**
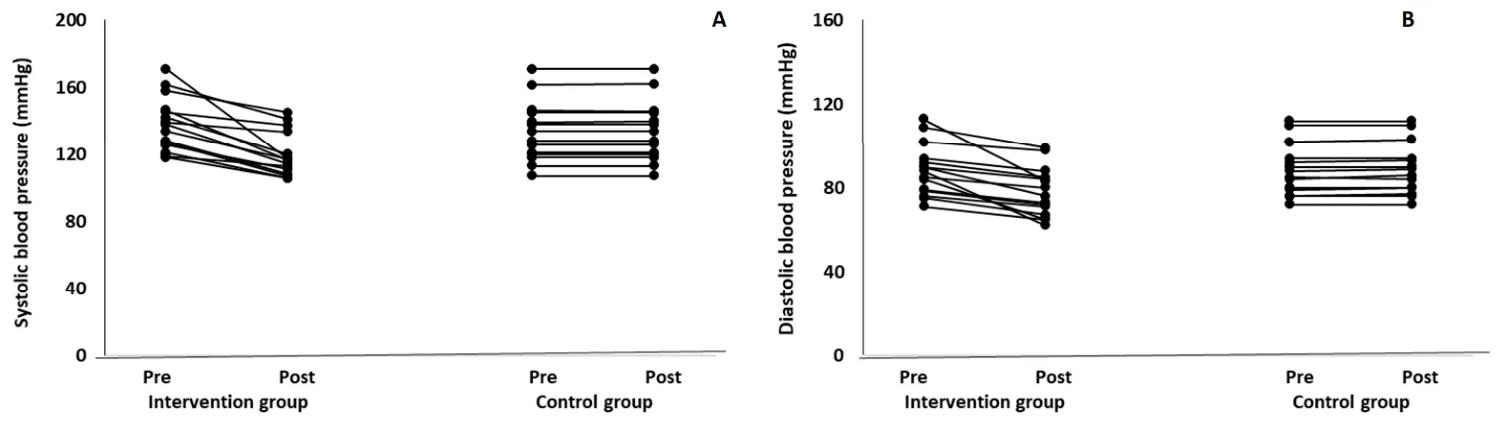
Individualized changes pre- and post-training in systolic pressure (**A**) and diastolic pressure (**B**) in the intervention and control groups.

When the results were analyzed and presented individually (Figure 5), we observed that all the participants in the IG, following the intervention program, reduced systolic and diastolic blood pressure values. The range of reduction was from −4.49 to −46.15% for the systolic pressure and from −4.08 to −41.94% for diastolic pressure, depending on the participant. In the CG, the individualized analyses indicated conflicting results (some participants reported no change, while others showed either a slight increase or decrease of blood pressure) following the 6 months.

#### 3.1.3. Respiratory Function (FVC and FEV_1_)

Repeated measures analyses of variances indicated significant “group × time” interaction effects on the FVC (*F*_1,34_ = 33.47; *p* < 0.001) and on the FEV_1_ (*F*_1,34_ = 45.58; *p* < 0.001). Specifically, in the IG, post-training values on the FVC (*p* < 0.05; Cohen *d* = 0.82 large effect size) and on the FEV_1_ (*p* < 0.05; Cohen *d* = 0.80 large effect size) were significantly higher compared to the pre-training measurements (Table 7). In CG, the above variables remained stable after 6 months (*p* = 0.86–0.87). Comparisons between groups revealed that pre-training FVC and FEV_1_ values were not different between groups (*p* = 0.98–0.99), but all post-training values were significantly higher in IG vs. CG (*p* < 0.05; Cohen *d* = 0.80–0.84 large effect size). Independent *t*-tests showed that the percentage changes (from baseline to final measurements) in FVC and FEV_1_ were significantly greater in IG than in CG (*p* < 0.001).

**Figure 6:**
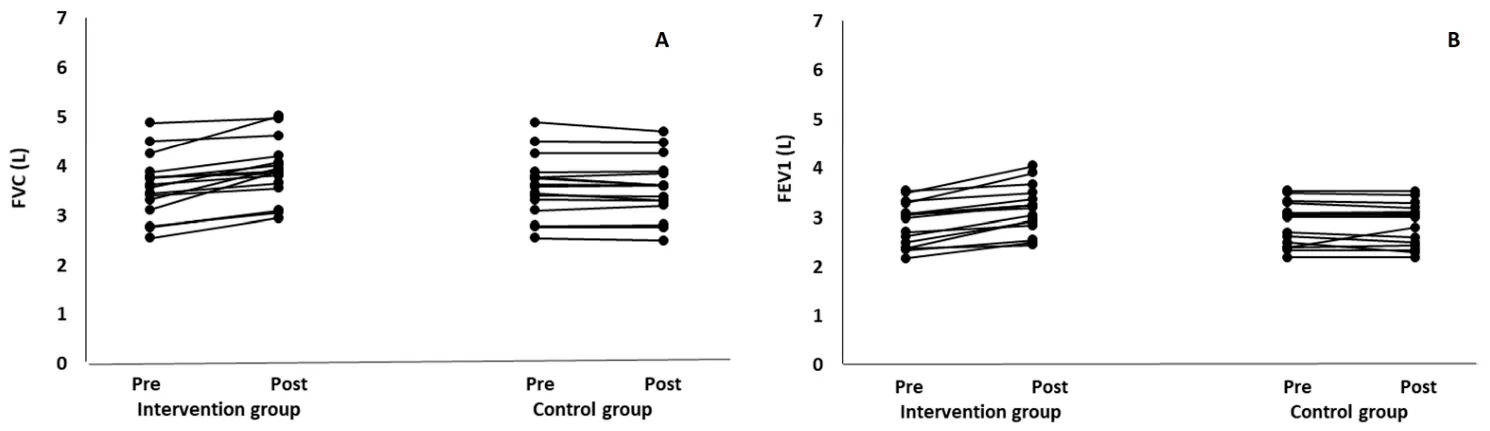
Individualized changes pre- and post-training in forced vital capacity—FVC (**A**) and forced expiratory volume in 1 s—FEV_1_ (**B**) in the intervention and control groups.

When the results were analyzed and presented individually (Figure 6), we observed that all the participants in the IG, following the intervention program, increased FVC and FEV_1_ values. The range of increase was from 1.82 to 20.26% for FVC and from 2.45 to 19.32% for FEV_1_, depending on the participant. In the CG, the individualized analyses indicated conflicting results (some participants reported no change, while others showed either a slight increase or decrease of FVC and FEV_1_) following the 6 months.

#### 3.1.4. Lipidemic Profile

Repeated measures analyses of variance indicated significant “group × time” interaction effects on the triglycerides (*F*_1,34_ = 28.17; *p* < 0.001), the total cholesterol (*F*_1,34_ = 23.07; *p* < 0.001), the HDL (*F*_1,34_ = 33.67; *p* < 0.001), and the LDL (*F*_1,34_ = 35.16; *p* < 0.001). Specifically, in IG, post-training triglycerides (*p* < 0.001; Cohen *d* = 1.06 large effect size), total cholesterol (*p* < 0.001; Cohen *d* = 1.54 large effect size), and LDL values (*p* < 0.001; Cohen *d* = 1.55 large effect size) were significantly lower compared to the pre-training values (Table 8). In contrast, in IG post-training, HDL values were significantly higher compared to pre-training values (*p* < 0.001; Cohen *d* = 1.20 large effect size). In CG, the above variables remained unchanged after 6 months (*p* = 0.92–0.99). Comparisons between groups revealed that pre-training lipidemic profile values were not different between groups (*p* = 0.75–0.99), but post-training triglycerides, total cholesterol, and LDL values were significantly lower in IG vs. CG, while post-training HDL values were significantly higher in IG vs. CG (*p* < 0.01; Cohen *d* = 1.05–1.69 large effect size). Independent *t*-tests showed that the percentage changes (from baseline to final measurements) in lipidemic profile values were significantly greater in IG than in CG (*p* < 0.001).

**Figure 7:**
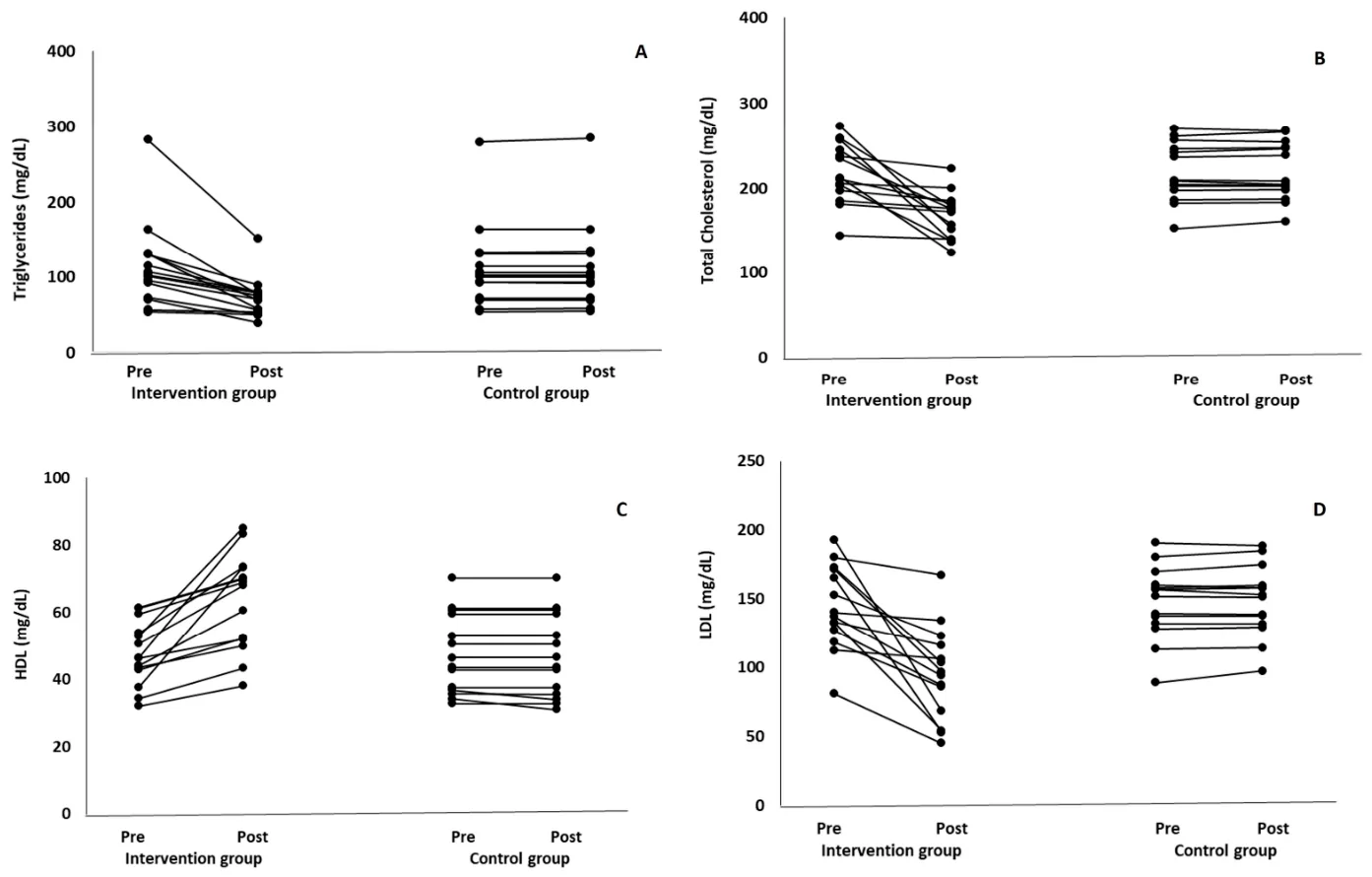
Individualized changes pre- and post-training in triglycerides (**A**), total cholesterol (**B**), high-density lipoprotein—HDL (**C**), and low-density lipoprotein—LDL (**D**) in the intervention and control groups.

When the results were analyzed and presented individually (Figure 7), we observed that all the participants in the IG, following the intervention program, reduced triglycerides, total cholesterol, and LDL values. The range of reduction was from −4.4 to −129.42% for triglycerides, from −2.50 to −87.59% for total cholesterol, and from −4.40 to −212.67% for LDL, depending on the participant. In contrast, all the participants in the IG increased HDL values (from 10.81 to 48.91%) following the intervention program. In the CG, the individualized analyses indicated conflicting results (some participants reported no change, while others showed either a slight increase or decrease) following the 6 months.

### 3.2. Functional Capacity Indices

#### 3.2.1. Flexibility (Sit-and-Reach Test), Static Balance (Single-Limb Stance Test), and Dynamic Balance (Timed Up-and-Go Test)

Repeated measures analyses of variance indicated significant “group × time” interaction effects on the sit-and-reach test (*F*_1,34_ = 82.17; *p* < 0.001), the single-limb stance test (right leg: *F*_1,34_ = 30.29, *p* < 0.001; left leg: *F*_1,34_ = 30.06, *p* < 0.001), and the timed up-and-go test (TUG test) (*F*_1,34_ = 29.65; *p* < 0.001). Specifically, in the IG, post-training values on the sit-and-reach (*p* < 0.001; Cohen *d* = 0.80 large effect size) and the single-limb stance test (right leg: *p* < 0.001, Cohen *d* = 1.91 large effect size; left leg: *p* < 0.001, Cohen *d* = 2.17 large effect size) were significantly higher compared to the pre-training measurements (Table 9). Moreover, TUG values (time in s) in the IG were significantly lower at post-training versus the pre-training measurements (*p* = 0.001; Cohen *d* = 0.81 large effect size). In CG, the above variables remained unchanged after 6 months (*p* = 0.28–0.99). Comparisons between groups revealed that pre-training flexibility and balance values were not different between groups (*p* = 0.77–0.99), but all post-training sit-and-reach and single-limb stance values were significantly higher in IG vs. CG (*p* < 0.001; Cohen *d* = 0.90–2.17 large effect size), whereas post-training TUG values were significantly lower in IG vs. CG (*p* < 0.001; Cohen *d* = 0.87 large effect size). Independent *t*-tests showed that the percentage changes (from baseline to final measurements) in flexibility and balance tests were significantly greater in IG than in CG (*p* < 0.001).

**Figure 8:**
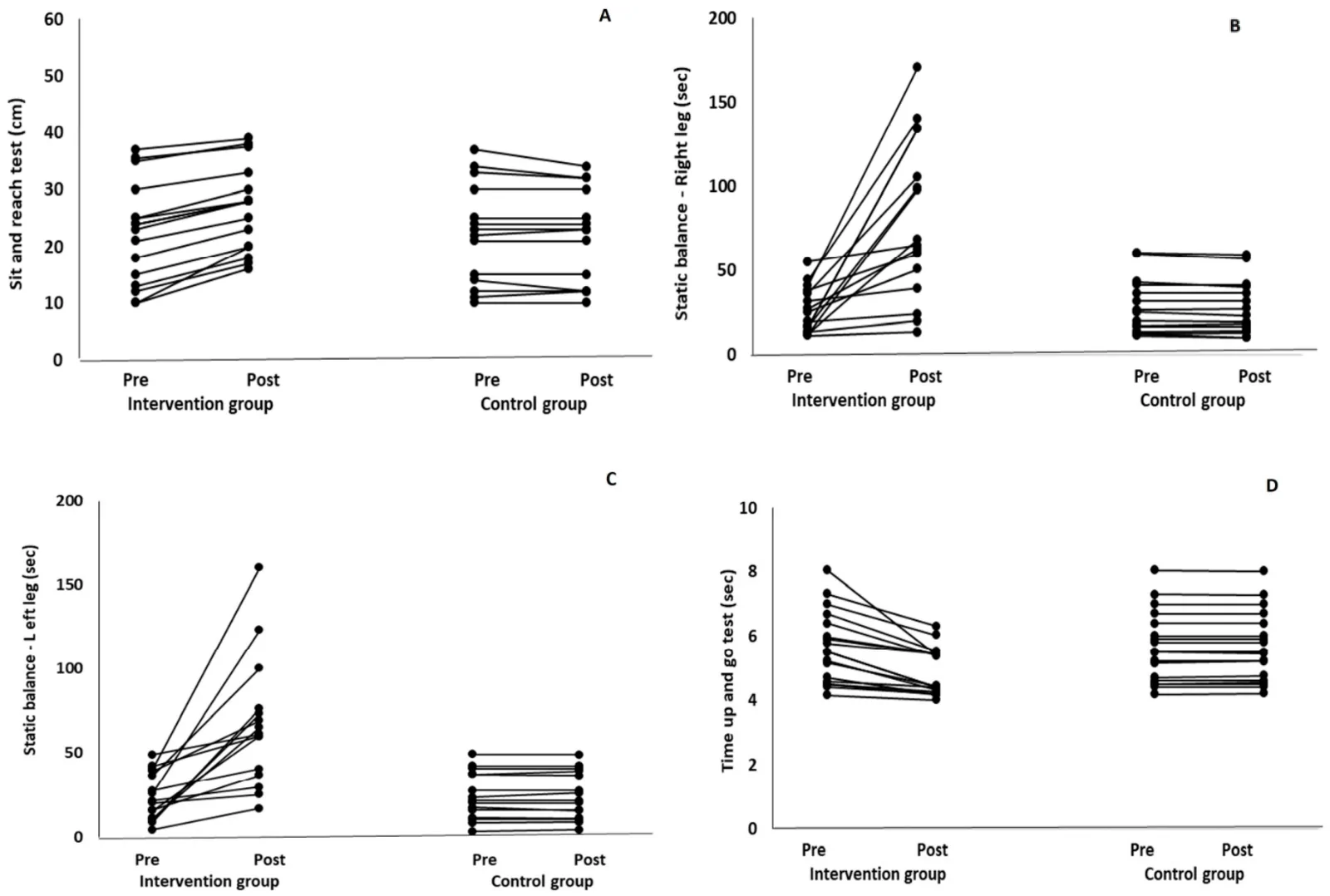
Individualized changes pre- and post-training in sit-and-reach test (**A**), single-stance test of the right leg (**B**), single-stance test of the left leg (**C**), and timed up-and-go test (**D**) in the intervention and control groups.

When the results were analyzed and presented individually (Figure 8), we observed that all the participants in the IG, following the intervention program, increased the sit-and-reach and the single-limb stance test values (for the right and left legs) and reduced the time in the TUG test. The range of increase in the sit-and-reach test was from 5.13 to 50%; the single-limb stance test was from 15.38 to 87.77% for the right leg and from 20.03 to 88.83% for the left leg, depending on the participant. Additionally, the range of reduction in time at the TUG test was from −3.75 to −48.07%, depending on the participant. On the other hand, in the CG, the individualized analyses indicated conflicting results (some participants reported no change; others showed either a slight increase or decrease) following the 6 months.

#### 3.2.2. Strength (Maximal Handgrip Strength Test)—Cardiovascular Fitness (YMCA Step Test)

Repeated measures analyses of variance indicated significant “group × time” interaction effects on the maximal handgrip strength of the right (*F*_1,34_ = 133.19; *p* < 0.001) and left (*F*_1,34_ = 317.39; *p* < 0.001) hands and on the heart rate after the termination of YMCA step test (*F*_1,34_ = 48.51; *p* < 0.001). Specifically, in the IG, post-training values on the handgrip strength test (right hand: *p* < 0.001; Cohen *d* = 0.81 large effect size; left hand: *p* < 0.001; Cohen *d* = 0.81 large effect size) were significantly higher compared to the pre-training measurements (Table 10). Moreover, heart rate values (1 min after the step test) in the IG were significantly lower at post-training versus the pre-training measurements (*p* = 0.001; Cohen *d* = 1.53 large effect size). In CG, the above variables remained unchanged after 6 months (*p* = 0.16–0.75). Comparisons between groups revealed that pre-training handgrip strength and heart rate values were not different between groups (*p* = 0.85–0.93), but all post-training handgrip strength values were significantly higher in IG vs. CG (*p* < 0.001; Cohen *d* = 0.87–0.88 large effect size), whereas post-training heart values were significantly lower in IG vs. CG (*p* < 0.001; Cohen *d* = 1.69 large effect size). Independent *t*-tests showed that the percentage changes (from baseline to final measurements) in handgrip strength and YMCA step tests were significantly greater in IG than in CG (*p* < 0.001).

**Figure 9:**
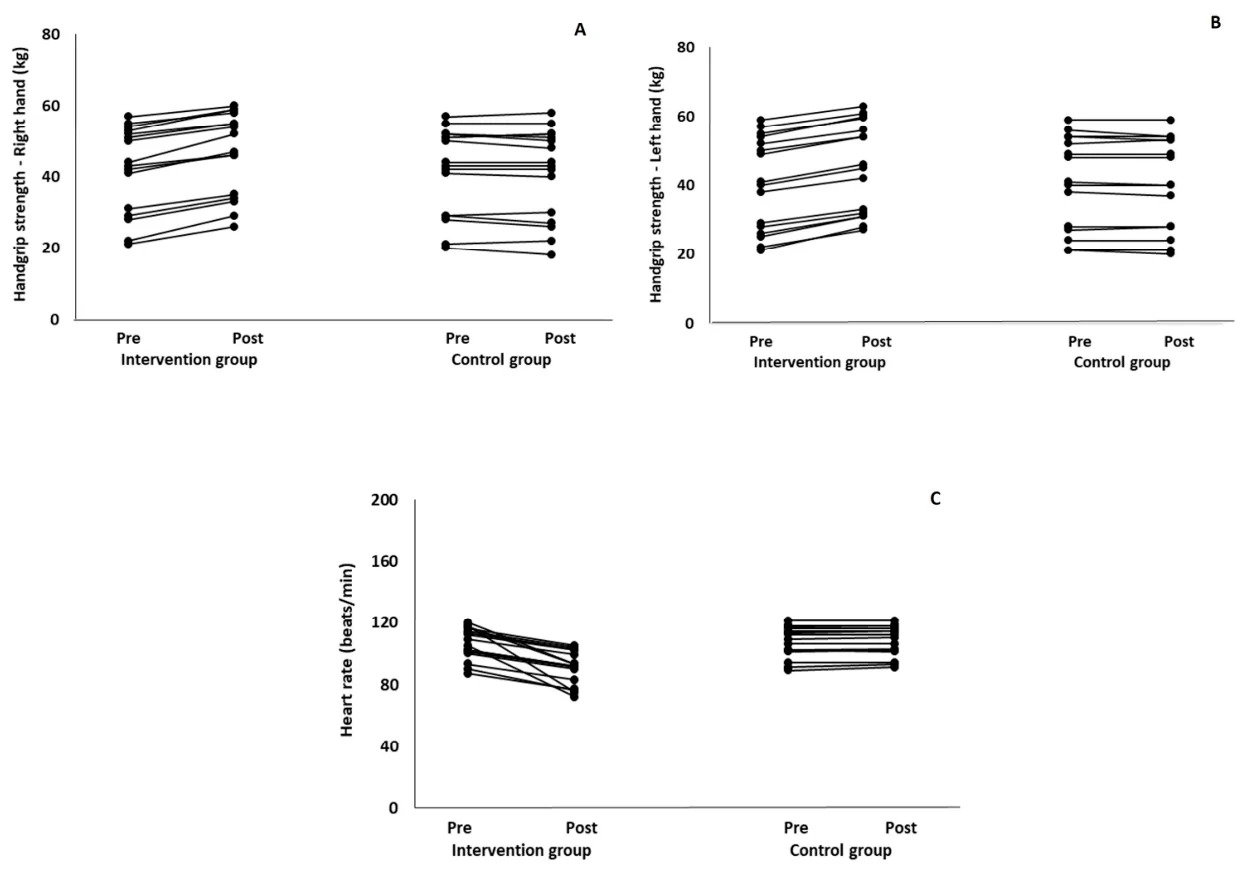
Individualized changes pre- and post-training in handgrip strength test of the right (**A**) and left hands (**B**) and heart rate 1 min after the termination of the step test (**C**) in the intervention and control groups.

When the results were analyzed and presented individually (Figure 9), we observed that all the participants in the IG, following the intervention program, increased the handgrip strength test values (for the right and left hands) and reduced the heart rate values 1 min after the step test. The range of increase in the handgrip strength test was from 5 to 24.4% for the right hand and from 6.35 to 25% for the left hand, depending on the participant. Additionally, the range of reduction in heart rate was from −9.62 to −57.33%, depending on the participant. On the other hand, in the CG, the individualized analyses indicated conflicting results (some participants reported no change, while others showed either a slight increase or decrease) following the 6 months.

### 3.3. Dietary Habits and Water Consumption

Repeated measures analyses of variance indicated significant “group × time” interaction effects on the MedDietScore (*F*_1,34_ = 132.76; *p* < 0.001) and the daily water consumption (*F*_1,34_ = 345.89; *p* < 0.001). Specifically, in the IG, post-training values on the MedDietScore were significantly higher compared to the pre-training measurements (*p* < 0.001; Cohen *d* = 1.26 large effect size) (Table 11). Additionally, daily water consumption values in the IG were significantly higher at post-training versus the pre-training measurements (*p* = 0.001; Cohen *d* = 2.35 large effect size). In CG, the above variables remained unchanged after 6 months (*p* = 0.26–0.44). Comparisons between groups revealed that pre-training MedDietScore and water consumption values were not different between groups (*p* = 0.88–0.95), but all post-training values were significantly higher in IG vs. CG (*p* < 0.001; Cohen *d* = 1.33–2.50 large effect size). Independent *t*-tests showed that the percentage changes (from baseline to final measurements) in MedDietScore and daily water consumption were significantly greater in IG than in CG (*p* < 0.001).

**Figure 10:**
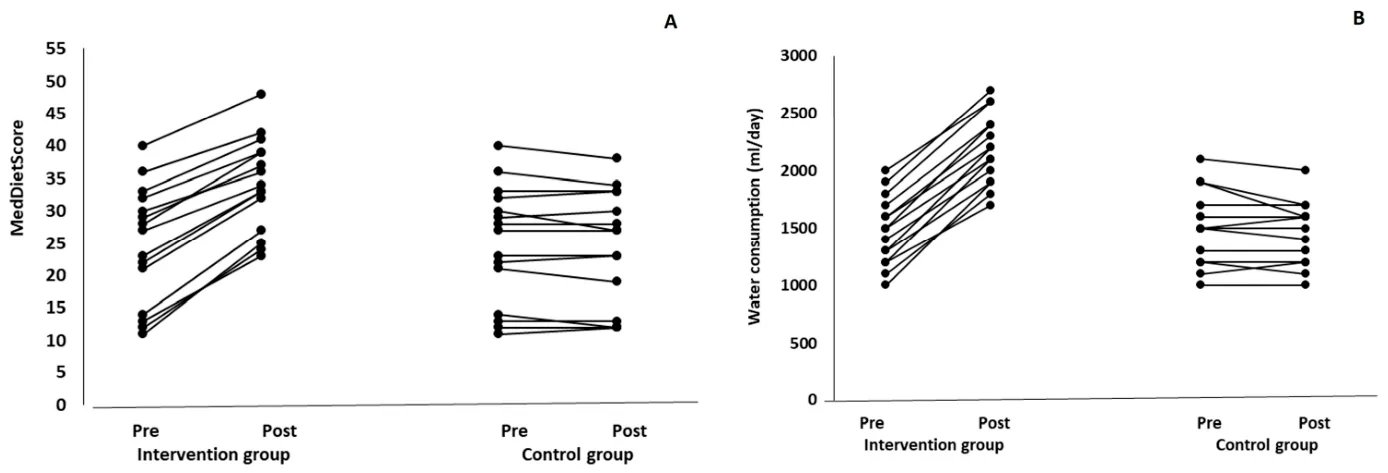
Individualized changes pre- and post-training in the Mediterranean Diet Score—MedDietScore (**A**) and the daily water consumption (**B**) in the intervention and control groups.

When the results were analyzed and presented individually (Figure 10), we observed that all the participants in the IG, following the intervention program, increased the MedDietScore and the daily water consumption. The range of increase in the MedDietScore was from 12.5 to 56% and from 23.08 to 50% for the water consumption, depending on the participant. On the other hand, in the CG, the individualized analyses indicated conflicting results (some participants reported no change, while others showed either a slight increase or decrease) following the 6 months.

## 4. Discussion

Our results demonstrated that a 6-month workplace combined intervention program (a) reduces body fat and circumferences, blood pressure, lipidemic profile (LDL, total cholesterol, and triglycerides) and heart rate recovery and (b) increases respiratory function, HDL, flexibility, static and dynamic balance, handgrip strength, MedDietScore, and water consumption, thus proving our research hypothesis. Although the program was effective for all the participants of the intervention group, a great variation in the magnitude of improvements was observed among the participants.

### 4.1. Responses of the Sample Mean Following the Intervention

One of the findings of the present study that is of primary importance is the significant reduction in systolic (mean change: −16.39 mmHg; −15.78%) and diastolic (mean change: −10.14 mmHg; −13.61%) blood pressures following the 6-month intervention program. According to the recommendations of the American College of Cardiology, the American Heart Association, and the World Health Organization, the reduction in blood pressure following a systematic exercise intervention may eliminate the risk of cardiovascular diseases [42,43]. Specifically, a systematic review and meta-analysis [44] demonstrated that every 10 mm Hg reduction in systolic blood pressure significantly reduces the risk of coronary heart disease, stroke, and heart failure, which leads to a significant 13% reduction in all-cause mortality. Another health parameter that showed significant change following the 6-month intervention program was lipidemic profile, where total cholesterol (mean change: 48.29 mg/dL; −31%), LDL (mean change: −52.57 mg/dL; −68.87%), and triglycerides (mean change: −40.92 mg/dL; −55.29%) decreased, while HDL (mean change: +15.17 mg/dL; +23.34%) increased following the intervention program. The average magnitude of improvement observed in our study is of vital importance, considering the results of previous meta-analysis [45] that reduction of LDL against 38.7 mg/dL (1 mmol/L) decrease the risk of cardiovascular mortality. Furthermore, in the present study, we also found significant reduction in body fat (mean change: −8.60%) and body circumferences (waist: mean change −8.67 cm, −8.76%; hip: mean change −10.5 cm, −9.97%) as well as significant increase of respiratory function (FVC: mean change +7.83%; FEV1: mean change +8.25%) following the 6-month intervention program.

The findings of the present study, regarding blood pressure and/or lipid profile indices, are differentiated from those of MacEwen et al. [25], which showed no change in cardiometabolic indices (blood pressure, LDL, and HDL total cholesterol) following a 12-week intervention (using sit-stand workstation) in office workers with abdominal obesity. In the same context, Phaswana et al. [26] investigated the efficiency of a 12-week height-adjustable sit-to-stand intervention on cardiometabolic health outcomes among overweight-obese office-based workers in South Africa, reporting small—trivial improvements. The different findings observed in our study compared to those of previous studies [25,26] may be attributed to the greater duration of intervention (6 months in the present study vs. 12 weeks in the MacEwen et al. and Phaswana et al. studies), the individual characteristics of the participants, as well as the intervention program (combined exercise emphasizing physical fitness parameters and nutrition intervention program in the present study vs. sit-stand workstation intervention in the MacEwen et al. and Phaswana et al. studies). On the other hand, the results of the present study are in line with a previous study [46] that showed significant reduction of LDL and total cholesterol following an 8-week intervention using stair climbing in overweight-obese office workers. Additionally, our findings are in line with previous studies that showed significant (a) reduction of body fat following a 6-month health program (unsupervised exercise sessions and monthly dietary/health education sessions) in overweight-obese office workers [30]; (b) reduction of waist (by 5.5 cm) and hip circumference (by 4.8 cm) following a 9-month intervention (using treadmill-workstation) in overweight-obese office workers [23]; and (c) increase in respiratory function following workplace exercise (aerobic capacity, strength, flexibility, and balance) interventions (from 6 weeks to 6 months) in office workers [41] and health professionals [47]. However, it should be mentioned that the percentage change of the body fat and circumferences, as well as of the respiratory function, following the intervention was greater in the present study compared to that observed in the above studies [23,30,41,47]. Differences in the baseline levels of health parameters, the sample characteristics, and the intervention characteristics may account for these equivocal findings regarding the magnitude of change among studies.

It should also be mentioned that the 6 months of the supervised combined workplace intervention program of the present study significantly altered different features of functional capacity and physical fitness such as flexibility (mean change: 21.65%), balance (mean change: 56.84–59.21%), handgrip strength (mean change: 10.56–11.32%), and heart rate 1 after the termination of step test (mean change: −18.13%). In the same context, a previous study of Ramli et al. [30] showed significant positive effects on maximum oxygen uptake, curl-up, and sit-and-reach test following a 6-month health program (including weekly unsupervised exercise sessions and monthly dietary/health education sessions) among overweight and obese office workers, while the push-up test remained unchanged. Moreover, our findings regarding functional capacity and physical fitness indices are in line with those of previous studies that performed exercise interventions and found significant improvement in different populations, such as physically active and normal weight office workers [48], healthcare employees working in a hospital environment [47], and overweight and/or obese women [12,13,14].

Additionally, an important aspect of this study is the significant increase (mean change: 30.98% in the IG) of the MedDietScore following the 6-month workplace combined intervention program. This is of crucial importance since previous studies showed that higher adherence to the Mediterranean diet is associated with a reduced risk of developing multiple chronic diseases and increased life expectancy [49]. In the present study, the adherence to the Mediterranean diet was assessed using the MedDietScore (total score ranges: from 0 to 55), where a score of 0 to 20 defines low compliance to the Mediterranean diet, a score of 21 to 35 defines moderate compliance, and a score of 36 to 55 defines high compliance [35,50]. It should be mentioned that at baseline measurements before the 6 months, the overwhelming majority of the participants in the IG demonstrated either low (33.33%) or moderate (55.55%) compliance with the Mediterranean diet, reinforcing the need for the design and implementation of appropriate interventions to improve dietary habits. Following the intervention program, we observed a significant mean increase of MedDietScore at 9.94 points, an important finding considering that a 2-point increase in adherence score to the Mediterranean diet is actually protective for health, reducing the occurrence of overall mortality (−8%), as well as the incidence of cardiovascular (−10%) and neoplastic diseases (−4%) [51]. In the present study, a significant increase in daily water consumption was also observed in the intervention group (34.43%) following the 6-month intervention program. This is also a significant finding since adequate water consumption is essential for cellular homeostasis and life [52], avoiding dehydration and its negative symptoms to the human body, such as unclear thinking, mood change, overheating, constipation, etc. The findings of the present study regarding the improvement of adherence to the Mediterranean diet are in line with a previous study [53] that investigated the efficacy of a 6-month “Stand Up for Healthy Aging” intervention (including sit-stand desks and motivational nudges), reporting higher adherence to the Mediterranean diet (using the MedDietScore) in the intervention group compared to the control group after the intervention. However, it should be mentioned that the percent change of the MedDietScore following the intervention was greater in our study compared to that observed in Santos et al. study [53]. Differences in the baseline level of MedDietScore of the intervention group and in the contents of the intervention may account for these equivocal findings regarding the magnitude of change between our study and that of Santos et al. [53].

### 4.2. Interpretation of Findings Observed from Individual Responses

In the present study from individual analyses of the data, it was observed that all the participants of the intervention group showed significant improvements in all examined indicators (health, functional capacity and physical fitness, and dietary habits); however, great variations in the magnitude of improvements were demonstrated among the participants. The findings of the present research are in line with those of a recent study [54], which demonstrated that a 6-month comprehensive exercise program in the workplace can lead to a significant reduction in musculoskeletal pain and an improvement in the functional capacity of office workers with musculoskeletal pains in different body parts, with great heterogeneity in the magnitude of the change following the intervention. The researchers of the above study [54] concluded that the magnitude of improvements is directly linked to differences in the initial level of pain and functional capacity, as well as to the participants’ adherence to the exercise program. In more detail, the participants who experienced longer duration and greater intensity of pain before the intervention exhibited greater improvement after the completion of the program [54]. Nevertheless, in the present study, the factors that could potentially affect the variation in individual responses following the intervention were not examined to draw safer and more reliable conclusions on this topic.

This observed differentiation in the magnitude of improvement following intervention programs acquires particular significance considering the contemporary methodological literature, which also points out that the comparison of mean values between groups may obscure functionally significant changes at the individual level [55]. According to Hays and Peipert [55], the evaluation of an intervention solely through group statistics can lead to an underestimation of its actual impact, especially when a significant percentage of participants show substantial improvement, which is offset by the stagnation or negative change of the others.

In the same context, the study by Mickan et al. [31], in which an educational intervention was evaluated in a small group of healthcare professionals, also demonstrates the importance of complementary interpretation through individual data. In the study of Mickan et al. [31], although the quantitative analyses showed limited or non-homogeneous changes in certain indicators at the mean level, the qualitative data highlighted substantial changes in perceptions, attitude, and self-efficacy in some of the participants. Thus, the authors emphasize that the effect of the intervention varied significantly according to individual factors, such as initial skill level, professional experience, and degree of active participation, thereby reinforcing the view that group analyses (using the mean values) alone do not sufficiently capture the complexity of responses to various interventions.

### 4.3. Limitations of the Study

The present study has some limitations that could affect its outcomes and, as a result, its generalization. An important limitation of this study is that, although the intervention of the present study was very effective for health promotion in obese office workers, its expended time, personnel, and resources for adherence to implementation is something that could affect its feasibility for duplication. For example, for some offices workers with increased responsibilities and without breaks during the working hours, maybe it is difficult to implement this program daily for 25–40 min per day. These employees, on some days, could implement this program before or after their work shift, or they could perform the program in small parts scattered throughout working hours.

The cost of the intervention (regarding the personnel used for the design and implementation of the intervention) is another limitation of this study. Furthermore, the present study does not examine factors that could potentially affect the variation in individual responses in order to draw more accurate findings regarding this interesting topic. Future studies, however, could examine possible factors (i.e., participants’ characteristics such as BMI value and initial level of measured indicators) that could affect the individual responses. Future studies could also investigate the efficacy of similar programs on various risk factors for type 2 diabetes or insulin resistance (including fasting glucose, Hb A1c, HOMA-IR, or oral glucose tolerance test).

Additional limitations of the present study are the mixed sample of males and females, the absence of categorization of the results considering the baseline fitness level, and the absence of a true placebo group. Future studies (with adequate sample sizes per sex and fitness level) could examine and compare possible differences in workplace interventions between males and females of various fitness levels. Also, during the study (before and after the 6 months), we only evaluated the adherence to the Mediterranean diet using the Mediterranean Diet Score, while we did not evaluate the actual food intake (i.e., total amount of calories, macronutrients, and micronutrients) of the participants. Moreover, in the present study, we did not evaluate important parameters such as workplace productivity, acute illness, and healthcare costs.

Finally, there is evidence that a safe and supportive work environment that actively promotes well-being is an essential duty of employers. A healthy workplace protects individuals from job-related hazards while proactively fostering optimal quality of life and sustained productivity. According to the WHO Healthy Workplace Framework, employers hold structural responsibilities across four distinct avenues: (a) physical environment (i.e., ergonomics, adequate lighting, and hazard mitigation); (b) psychosocial environment (i.e., managing workload demands and preventing workplace bullying/harassment); (c) work organization and job design (i.e., ensuring clear expectations, reasonable working hours, and predictable schedules); and (d) personal health and community connections (i.e., offering equitable health benefits, supporting healthy habits, and providing family-supportive policies). In our study, we have not looked into something like that; however, a future study could emphasize this holistic approach regarding the employers’ duties.

## 5. Conclusions

In summary, the daily 6-month supervised workplace combined intervention program (exercise and nutrition) decreased selected health risk factors, such as blood pressure, lipidemic profile, body mass, body fat, and body circumferences in obese office workers. Additionally, the program increased selected functional capacity and physical fitness indicators and improved adherence to the Mediterranean diet and daily water consumption. Although all the participants of the intervention group showed improvements on the evaluated parameters, the analysis of individual data revealed substantial heterogeneity in the magnitude of responses. This heterogeneity in the individual responses reinforces the importance of complementary evaluation of data at the individual level in future studies.

## Figures and Tables

**Table 1:** Demographic, anthropometric, and working characteristics of the participants per group (mean ± standard deviation).

Characteristics	IG (*n* = 18)	CG (*n* = 18)
Demographic characteristics		
Age (years old)	48.06 ± 6.60	47.95 ± 5.95
Sex	11 females, 7 males	11 females, 7 males
Anthropometric characteristics		
Body mass (kg)	95.65 ± 11.88	95.54 ± 11.60
Body height (m)	1.69 ± 0.11	1.68 ± 0.10
BMI (kg/m^2^) *	33.57 ± 1.58	33.54 ± 1.74
Working characteristics		
Working experience (years)	18.00 ± 7.00	17.50 ± 7.50
Working hours/day	8.90 ± 0.20	8.80 ± 0.20
Working hours/week (hours)	44.50 ± 2.50	44.00 ± 2.50

* BMI: body mass index = body mass/body height^2^; IG: intervention group; CG: control group.

**Table 2:** Indicative weekly tables with the exercise goals of the intervention program.

Weekly Exercise Goals for Week 3–4				
Monday	Tuesday	Wednesday	Thursday	Friday
Flexibility Strength _(c,d)_ Aerobic capacity	Strength _(a,b)_ Balance	Flexibility Aerobic capacity	Balance Strength _(c,d)_	Flexibility Strength _(a,b)_ Aerobic capacity
**Weekly Exercise Goals for Week 17–19**				
**Monday**	**Tuesday**	**Wednesday**	**Thursday**	**Friday**
Flexibility Strength _(a,b,c,d)_ Aerobic capacity	Flexibility Balance Aerobic capacity	Flexibility Balance Strength _(a,c)_	Flexibility Strength _(b,d)_ Aerobic capacity	Flexibility Strength _(a,c)_ Balance Aerobic capacity

Strength a: core strength exercises (lower and upper back, chest, and abdominal exercises). Strength b: exercises for upper limbs and hands. Strength c: strength exercises for the cervical joint (neck exercises). Strength d: strength exercises for lower limbs.

**Table 3:** Training contents and variables of the exercise program.

Exercise Goals	Exercise Contents—Activities	Exercise Variables
Flexibility	✓ Static stretching exercises ✓ Dynamic exercises ✓ Mind-body exercises (Tai chi, Yoga, Pilates)	✓ Frequency: 3–5 times/week (each muscle group or joint) ✓ Set/exercise: 1–2 ✓ Duration—Repetitions (reps)/set: 10–20 s (in static exercises) or 10–20 reps (in dynamic exercises)
Balance	✓ Static balance exercises using bipedal and monopedal stance ✓ Dynamic balance exercises (heel walking, toe walking, tandem walking, side walking, crossover walking, etc.) ✓ Mind-body exercises (Tai chi and Yoga exercises)	✓ Frequency: 2–3 times/week ✓ Set/exercise: 1–2 ✓ Duration—Reps—Distance/set: 10–30 s—8–20 reps—3–6 m
Strength	✓ Strength exercises for (a) core, (b) upper limbs and hands, (c) cervical spine, and (d) lower limbs	✓ Frequency: 2–3 times/week (each muscle group or joint) ✓ Set/exercise: 1–2 ✓ Repetitions/set: 8–15 RM
Aerobic capacity	✓ Low-impact aerobic dance movements from a sitting position in a chair or from an upright position around the chair ✓ During the aerobic dance, there was continuous arm movement at the shoulder level as well as above the head.	✓ Frequency: 3–4 times/week ✓ Intensity: 60–80% of maximum heart rate (HRmax) * ✓ Music rhythm: 110–120 beats/min ✓ Duration/day: 14–20 min

* HRmax: maximum heart rate; Reps: repetitions; RM: repetitions maximum. Heart rate was measured using a specialized heart rate monitor, Polar H10 (Polar, Kempele, OUL, Finland). The participants wore a chest strap with the transmitter positioned on top. The transmitter was connected to a tablet application, Polar Team Solution system (Polar, Kempele, OUL, Finland), providing the participant’s heart rate at any given moment (real-time monitoring).

**Table 4:** Contents of the 12 sessions.

Session	Contents per Session
1 (group session; duration: 30 min)	Presentation regarding healthy diet and proper hydration and sharing of educational material (in printed form) for healthy diet to the participants of the intervention group.
2 (group session; duration: 25 min)	(a) Installation and familiarization with the “Waterful” application; (b) calculation of the required amount of water per day for each participant, and (c) setting of individual goals for increasing water intake using a goal-setting card.
3 (individual session; duration: 20 min/participant)	Meeting, with each participant of the intervention group, to provide some individual advice and set personal goals depending on the initial evaluation of the Mediterranean diet score.
4–12 (group sessions; duration: 25 min)	Discussion—Goal tracking regarding nutrition and hydration—Setting goals for the next weeks.

**Table 5:** Body mass, body fat, and body circumference values in the intervention and control groups pre-and post-training (mean ± SD).

Variables	Group	Pre-Training	Post-Training	Mean % Change
Body mass (kg)	IG	95.65 ± 11.88	90.34 ± 11.32 *^§^	−5.56 ± 0.88 ^†^
	CG	95.54 ± 11.60	95.94 ± 11.55	+0.45 ± 1.18
Body fat (%)	IG	46.66 ± 3.31	38.06 ± 2.49 *^#^	−8.60 ± 1.11 ^†^
	CG	46.64 ± 3.20	46.89 ± 2.94	+0.24 ± 0.99
Waist circumference (cm)	IG	106.78 ± 8.49	98.11 ± 6.32 *^#^	−8.76 ± 2.26 ^†^
	CG	106.83 ± 8.49	107.39 ± 8.30	+0.53 ± 1.02
Hip circumference (cm)	IG	116.67 ± 5.65	106.17 ± 5.59 *^#^	−9.97 ± 3.59 ^†^
	CG	115.71 ± 7.23	116.22 ± 7.03	+0.44 ± 0.91

CG: control group; IG: intervention group. Where * *p* < 0.001 is the statistically significant difference between the pre- and post-intervention programs in IG, ^#^ *p* < 0.001 is the statistically significant difference between IG and CG in the post-training measurement, ^§^ *p* < 0.05 is the statistically significant difference between IG and CG in the post-training measurement, and ^†^ *p* < 0.001 is the statistically significant difference between IG and CG in the percent change.

**Table 6:** Systolic and diastolic blood pressure values in the intervention and control groups pre- and post-training (mean ± SD).

Variables	Group	Pre-Training	Post-Training	Mean % Change
Systolic BP (mmHg)	IG	137.83 ± 16.92	121.44 ± 14.16 *^#^	−15.78 ± 9.99 ^†^
	CG	135.67 ± 18.03	136.75 ± 17.27	+1.04 ± 0.34
Diastolic BP (mmHg)	IG	88.03 ± 11.45	77.89 ± 10.74 *^#^	−13.61 ± 11.10 ^†^
	CG	88.33 ± 11.11	88.72 ± 11.09	+0.44 ± 0.81

BP: blood pressure; CG: control group; IG: intervention group. Where * *p* < 0.001 is the statistically significant difference between the pre- and post-intervention programs in IG, ^#^ *p* < 0.001 is the statistically significant difference between IG and CG in the post-training measurement, and ^†^ *p* < 0.001 is the statistically significant difference between IG and CG in the percent change.

**Table 7:** Respiratory function values in the intervention and control groups pre- and post-training (mean ± SD).

Variables	Group	Pre-Training	Post-Training	Mean % Change
FVC (L)	IG	3.70 ± 0.36	4.01 ± 0.40 *^#^	+7.83 ± 5.63 ^†^
	CG	3.71 ± 0.40	3.70 ± 0.35	−0.17 ± 0.53
FEV_1_ (L)	IG	3.03 ± 0.34	3.31 ± 0.36 *^#^	+8.25 ± 4.96 ^†^
	CG	3.04 ± 0.39	3.03 ± 0.34	−0.09 ± 0.92

FVC: Forced vital capacity; FEV_1_: forced expiratory volume in 1 s; CG: control group; IG: intervention group. Where * *p* < 0.001 is the statistically significant difference between the pre- and post-intervention programs in IG, ^#^ *p* < 0.05 is the statistically significant difference between IG and CG in the post-training measurement, and ^†^ *p* < 0.001 is the statistically significant difference between IG and CG in the percent change.

**Table 8:** Lipidemic profile values in the intervention and control groups pre- and post-training (mean ± SD).

Variables	Group	Pre-Training	Post-Training	Mean % Change
Triglycerides (mg/dL)	IG	113.05 ± 52.27	72.13 ± 25.02 *^#^	−55.29 ± 34.75 ^†^
	CG	113.19 ± 52.62	113.37 ± 52.95	0.11 ± 0.95
Total cholesterol (mg/dL)	IG	217.94 ± 36.93	169.65 ± 25.82 *^#^	−31.00 ± 29.34 ^†^
	CG	218.68 ± 35.54	219.41 ± 34.42	0.42 ± 1.66
HDL (mg/dL)	IG	45.72 ± 10.12	60.89 ± 15.19 *^#^	23.43 ± 11.68 ^†^
	CG	46.93 ± 11.43	46.96 ± 11.47	0.05 ± 0.23
LDL (mg/dL)	IG	149.48 ± 34.49	96.91 ± 33.30 *^#^	−68.87 ± 62.69 ^†^
	CG	149.11 ± 30.78	149.77 ± 29.26	0.66 ± 2.51

CG: control group; HDL: high-density lipoprotein; IG: intervention group; LDL: low-density lipoprotein. Where * *p* < 0.001 is the statistically significant difference between the pre- and post-intervention programs in IG, ^#^ *p* < 0.05 is the statistically significant difference between IG and CG in the post-training measurement, and ^†^ *p* < 0.001 is the statistically significant difference between IG and CG in the percent change.

**Table 9:** Flexibility and balance (static and dynamic) values in the intervention and control groups pre- and post-training (mean ± SD).

Variables	Group	Pre-Training	Post-Training	Mean % Change
Sit-and-reach test (cm)	IG	21.03 ± 6.23	25.81 ± 5.66 *^#^	21.65 ± 13.63 ^†^
	CG	21.39 ± 5.51	20.94 ± 5.13	−2.22 ± 5.80
Single-limb stance test-RL (s)	IG	26.27 ± 12.98	81.71 ± 45.12 *^#^	56.84 ± 27.07 ^†^
	CG	27.71 ± 15.55	27.06 ± 14.95	−2.51 ± 6.58
Single-limb stance test-LL (s)	IG	23.93 ± 12.91	78.55 ± 37.41 *^#^	59.21 ± 24.12 ^†^
	CG	23.84 ± 12.97	23.94 ± 12.92	1.45 ± 7.35
Timed up-and-go test (s)	IG	5.63 ± 1.12	4.88 ± 0.73 *^#^	−14.94 ± 10.88 ^†^
	CG	5.63 ± 1.11	5.64 ± 1.00	0.12 ± 0.51

IG: intervention group; CG: control group; RL: right leg; LL: left leg. Where * *p* < 0.001 is the statistically significant difference between the pre- and post-intervention programs in IG, ^#^ *p* < 0.001 is the statistically significant difference between IG and CG in the post-training measurement, and ^†^ *p* < 0.001 is the statistically significant difference between IG and CG in the percent change.

**Table 10:** Handgrip strength and heart rate values in the intervention and control groups pre- and post-training (mean ± SD).

Variables	Group	Pre-Training	Post-Training	Mean % Change
Handgrip strength-RH (kg)	IG	43.72 ± 5.45	48.33 ± 5.93 *^#^	10.56 ± 5.37 ^†^
	CG	43.22 ± 6.55	42.78 ± 6.83	−1.51 ± 4.10
Handgrip strength-LH (kg)	IG	41.67 ± 5.66	46.36 ± 5.92 *^#^	11.32 ± 5.24 ^†^
	CG	41.28 ± 7.23	41.01 ± 6.12	−0.66 ± 2.02
HR-1 min after test (beats/min)	IG	106.78 ± 9.92	91.11 ± 10.49 *^#^	−18.13 ± 13.63 ^†^
	CG	107.39 ± 9.68	107.92 ± 9.37	0.52 ± 0.86

IG: intervention group; CG: control group; HR: heart rate; RH: right hand; LH: left hand. Where * *p* < 0.001 is the statistically significant difference between the pre- and post-intervention programs in IG, ^#^ *p* < 0.001 is the statistically significant difference between IG and CG in the post-training measurement, and ^†^ *p* < 0.001 is the statistically significant difference between IG and CG in the percent change.

**Table 11:** Mediteranean Diet Score (MedDietScore) and daily water consumption in the intervention and control groups pre- and post-training (mean ± SD).

Variables	Group	Pre-Training	Post-Training	Mean % Change
MedDietScore (Scale 0–55)	IG	24.50 ± 9.01	34.44 ± 6.80 *^#^	30.98 ± 14.56 ^†^
	CG	25.00 ± 8.88	24.00 ± 8.90	−2.43 ± 6.66
Water consumption (mL/day)	IG	1422.22 ± 309.75	2155.56 ± 314.78 *^#^	34.43 ± 7.31 ^†^
	CG	1438.89 ± 329.24	1405.56 ± 285.89	−2.06 ± 6.33

MedDietScore: Mediterranean diet score; IG: intervention group; CG: control group. Where * *p* < 0.001 is the statistically significant difference between the pre- and post-intervention programs in IG, ^#^ *p* < 0.001 is the statistically significant difference between IG and CG in the post-training measurement, and ^†^ *p* < 0.001 is the statistically significant difference between IG and CG in the percent change.

## Data Availability

Data are contained within the article.

## Supplementary Materials

The following supporting information can be downloaded at: https://www.mdpi.com/article/10.3390/jfmk11040418/s1. Table S1: Eleventh exercise session of the 6-month workplace program; Table S2: Ninety-first exercise session of the 6-month workplace program; Table S3: CONSORT checklist.
