# Supplementary material for: A Combined Workplace Intervention Including Exercise and Nutrition for Health Promotion in Obese Office Workers: A Randomized Controlled Study
Source: J Funct Morphol Kinesiol. 2026 Oct 13;11(4):418. doi: 10.3390/jfmk11040418 (PMC13692482; doi:10.3390/jfmk11040418)
Supplement: Supplementary file 1 [file jfmk-11-00418-s001.zip › jfmk-4568618-supplementary.pdf]

Supplementary Table S1. 11th exercise session of the 6-month workplace program.

Total Duration of the 11th Training Session: 34 min

Warm-up (3 min): Low-impact chair-based seated aerobic dance movements in conjunction with dynamic movements of upper limbs (below and above the shoulder level).

Main part (28 min)

Part 1. Flexibility (4 min): static stretching exercises (1 set x 10 s per exercise)

Exercise 1 | Exercise 2 | Exercise 3 | Exercise 4 | Exercise 5

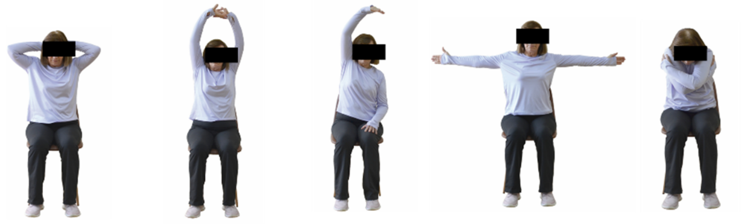

Exercise 6 | Exercise 7 | Exercise 8 | Exercise 9

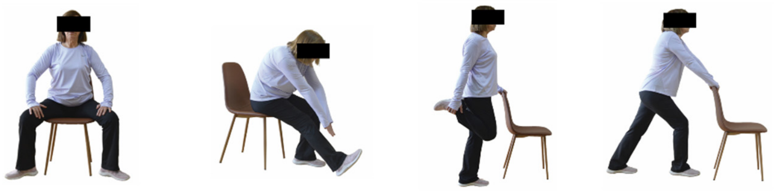

Part 2. Strength of cervical joint and lower limbs (10 min)

Cervical isometric strength exercises (2 min): (A) extension, (B) forward flexion and (C) lateral flexion and (D) lateral turn (2 sets × 3 s isometric contraction per exercise, with 10 s rest among sets and exercises).

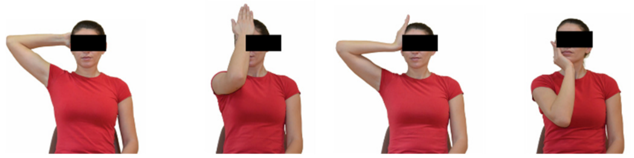

Lower limb strength exercises (8 min): (A) adductor mini ball squeeze from sitting position, (B) sit-to-stand exercise, (C) leg abduction from standing position and (D) leg flexion from standing position (2 sets x 8 reps per exercise, 20-25 sec rest among sets and exercises)

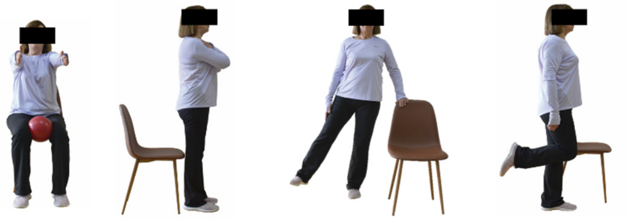

Part 3. Chair-based aerobic dance (14 min)

Low impact aerobic dance movements from a sitting position in a chair (i.e. march, step touch, knee lift, kick, V-step). During the aerobic dance there was continuous arm movement at the shoulder level and below that. Training characteristics: a) 65% of HRmax, b) music rhythm: 110-115 beats/min and c) duration: 14 min.

Cool down (3 min): Static stretching for the whole body in conjunction with breathing exercises.

*HRmax: maximum heart rate; reps: repetitions.

Supplementary Table S2. 91st exercise session of the 6-month workplace program.

Total Duration of the 91st Training Session: 40 min

Warm-up (3 min): Low-impact chair-based seated aerobic dance movements in conjunction with dynamic movements of upper limbs (below and above the shoulder level).

Main part (34 min)

Part 1. Flexibility (6 min): static and dynamic stretching exercises
(1 set x 20 reps for dynamic exercises or 20 s for static exercises)

Dynamic stretching exercises

Exercise 1

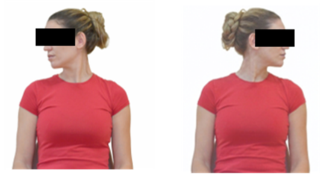

Exercise 2

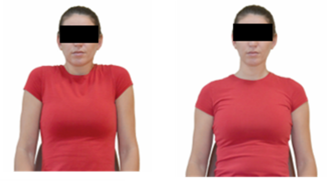

Exercise 3

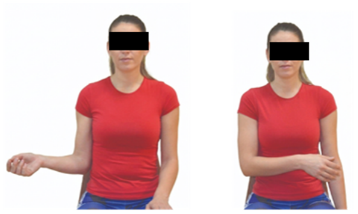

Exercise 4

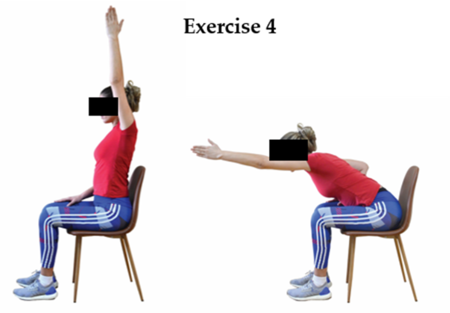

Static stretching exercises

Exercise 5

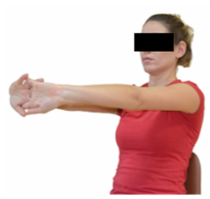

Exercise 6

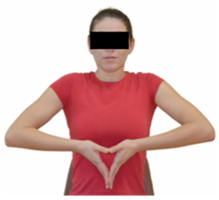

Exercise 7

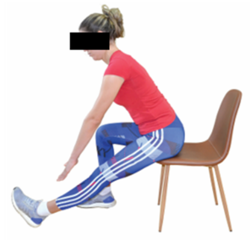

Exercise 8

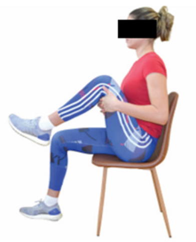

Exercise 9

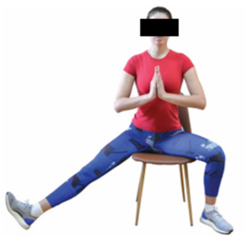

Exercise 10

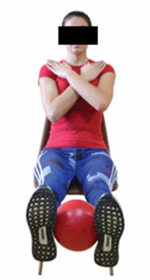

Part 2. Integrated combined aerobic and strength training program (28 min): during this part of the program that included seven small blocks of approximately 4 min (without rest between blocks), strength and aerobic training were altered in a predetermined order (2.5-min aerobic dance at 70-75% of HRmax/1.5-min strength exercises).

Block 1. Seated aerobic dance with continuous hand movements using a mini ball (2.5 min) / 2 strength exercises: (A) squat with hands forward (at shoulder level), pressing a mini ball (1 set × 12 reps) and (B) calf raises with simultaneous triceps extension pressing a min ball (1 set × 12 reps).

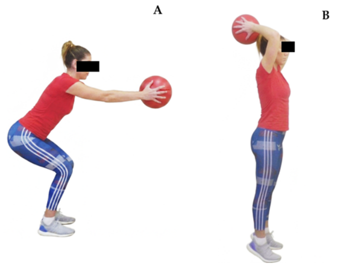

Block 2. Block 1 was repeated.

Block 3. Standing aerobic dance with continuous hand movements using a pair of hand grippers (2.5 min) / 2 strength exercises: (A) alternate leg abduction with simultaneous lateral hand raises (at shoulder level) squeezing hand grippers (1 set × 12 reps) and (B) lateral sit-ups from sitting position (1 set × 12 reps).

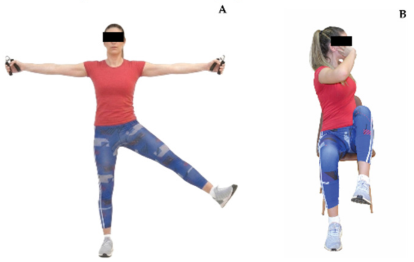

Block 4. Block 3 was repeated.

Block 5. Seated aerobic dance with continuous hand movements using mini ball (2.5 min) / 2 strength exercises: (A) seated cat-cow with adductor squeeze using a mini ball (1 set × 12 reps) and (B) alternate knee flexion from sitting position with simultaneous raising of the hands above the head squeezing a min ball (1 set × 12 reps).

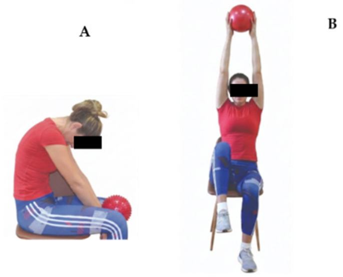

Block 6. Block 5 was repeated.

Block 7. Seated aerobic dance with continuous hand movements (2.5 min)/3 cervical isometric strength exercises with exercise band: extension, forward flexion and lateral flexion (4 sets × 6 s isometric contraction per exercise).

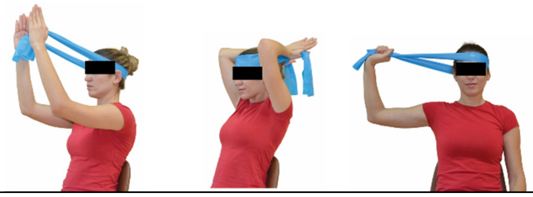

Cool down (3 min): Static stretching for the whole body in conjunction with breathing exercises.

*HRmax: maximum heart rate; reps: repetitions.

Supplementary Table S3. CONSORT checklist

Section/Topic	Item No	Checklist item	Reported on page No
**Title and abstract**			
	1a	Identification as a randomised trial in the title	Page 1: title
	1b	Structured summary of trial design, methods, results, and conclusions	Page 1: lines 14-34
**Introduction**			
Background and objectives	2a	Scientific background and explanation of rationale	Pages 1-3; lines 39-125
	2b	Specific objectives or hypotheses	Page 3; lines 106-114 and 126-132
**Methods**			
Trial design	3a	Description of trial design (such as parallel, factorial) including allocation ratio	Page 4 Figure 1/ Page 12; lines 250-256
	3b	Important changes to methods after trial commencement (such as eligibility criteria)	Page 4; lines 145-150
Participants	4a	Eligibility criteria for participants	Page 4; lines 145-150
	4b	Settings and locations where the data were collected	Page 12; lines 242-245
Interventions	5	The interventions for each group with sufficient details to allow replication, including how and when they were actually administered	Pages 5-10
Outcomes	6a	Completely defined pre-specified primary and secondary outcome measures, including how and when they were assessed	Page 11
	6b	Any changes to trial outcomes after the trial commenced, with reasons	N/A
Sample size	7a	How sample size was determined	Page 12; lines 276-281
	7b	When applicable, explanation of any interim analyses and stopping guidelines	N/A
Randomisation:			
Sequence generation	8a	Method used to generate the random allocation sequence	Page 12; lines 250-256
	8b	Type of randomisation; details of any restriction (such as blocking and block size)	Page 12; lines 250-256

Allocation concealment mechanism	9	Mechanism used to implement the random allocation sequence (such as sequentially numbered containers), describing any steps taken to conceal the sequence until interventions were assigned	Page 12; lines 250-256
Implementation	10	Who generated the random allocation sequence, who enrolled participants, and who assigned participants to interventions	Page 12; lines 250-256
Blinding	11a	If done, who was blinded after assignment to interventions (for example, participants, care providers, those assessing outcomes) and how	Page 12; lines 256-259
	11b	If relevant, description of the similarity of interventions	N/A
Statistical methods	12a	Statistical methods used to compare groups for primary and secondary outcomes	Pages 12-13; lines 281-290
	12b	Methods for additional analyses, such as subgroup analyses and adjusted analyses	N/A
**Results**			
Participant flow (a diagram is strongly recommended)	13a	For each group, the numbers of participants who were randomly assigned, received intended treatment, and were analysed for the primary outcome	Page 4 Figure 1
	13b	For each group, losses and exclusions after randomisation, together with reasons	Page 4 Figure 1
Recruitment	14a	Dates defining the periods of recruitment and follow-up	N/A
	14b	Why the trial ended or was stopped	N/A
Baseline data	15	A table showing baseline demographic and clinical characteristics for each group	Pages 5 Table 1
Numbers analysed	16	For each group, number of participants (denominator) included in each analysis and whether the analysis was by original assigned groups	Page 4 Figure 1
Outcomes and estimation	17a	For each primary and secondary outcome, results for each group, and the estimated effect size and its precision (such as 95% confidence interval)	Pages 13-21
	17b	For outcomes, presentation of effect sizes is recommended	Pages 13-21
Ancillary analyses	18	Results of any other analyses performed, including subgroup analyses and adjusted analyses, distinguishing pre-specified from exploratory	N/A
**Discussion**	19		22-25
**Other information**			

Registration	23	Registration number and name of trial registry	The study was registered at ClinicalTrials.gov (Identifier: NCT07603141)
Protocol	24	Where the full trial protocol can be accessed, if available	https://clinicaltrials.gov/study/NCT07603141
Funding	25	Sources of funding and other support (such as supply of drugs), role of funders	No funding
